# Supplementary material for: VerteBrain reveals novel neural and non-neural protein assemblies conserved across vertebrate evolution
Source: bioRxiv. 2025 May 28:2025.05.26.656196. Preprint. [Version 1] doi: 10.1101/2025.05.26.656196 (PMC12154601; doi:10.1101/2025.05.26.656196)
Supplement: Supplementary file 1 [file NIHPP2025.05.26.656196V1-supplement-1.pdf]

bioRxiv preprint doi: https://doi.org/10.1101/2025.05.26.656196; this version posted May 28, 2025. The copyright holder for this preprint (which was not certified by peer review) is the author/funder, who has granted bioRxiv a license to display the preprint in perpetuity. It is made available under aCC-BY 4.0 International license.

SUPPLEMENTAL FIGURE LEGENDS

Figure S1. Alphafold-multimer score distributions for sampled protein pairs as a function of CF-MS interaction score.

Figure S2. Co-elution profiles show intact subunits of the CCT chaperone and COP9 signalosome complexes from CF-MS experiments in zebrafish brains. Elution profiles were derived from ion exchange chromatography (IEX) followed by size exclusion chromatography (SEC). Each line represents the abundance of an individual complex subunit of the CCT chaperone and COP9 signalosome complexes across chromatographic fractions. The consistent co-elution of all annotated subunits within each complex indicates intact assembly and validates the resolution and reliability of the CF-MS workflow in adult zebrafish brain lysates.

Figure S3. Area under the receiver operating characteristic curve (AUROC) values for correctly associating proteins in VerteBrain that are known to belong to the same Gene Ontology (GO) Protein containing complex or Cellular anatomical entity. Leave-one-out network propagation analysis (see **Methods**) was used to evaluate how well protein-protein interaction scores from the VerteBrain dataset recapitulate known protein co-memberships in Gene Ontology (GO) categories. Left: AUROC values for proteins annotated as part of the same GO cellular component term categorized as a "Protein-containing complex" (n = 360 terms). Right: AUROC values for GO terms categorized as "Cellular anatomical entity" (n = 494 terms). High AUROC values indicate strong agreement between predicted interactions in VerteBrain and established GO co-complex or co-localization annotations. Dotted vertical lines indicate the baseline of random performance (AUROC = 0.5)

Figure S4. Enrichment plots for selected significant (FDR < 1%) interactors identified from IP-MS experiments in mouse whole brain lysates. Each plot reports mass spectrometry-measured abundances (colored dots) of the prey protein labeled in the top left of the plot following immunoprecipitation from whole mouse brain with antibodies targeting the bait protein labeled on the x-axis, accompanied by data from matched IP experiments using antibodies targeting either GFP or FLAG as negative controls. Abundances are reported in normalized counts per million (cpm) as measured using Degust (see **Methods**) across 3-4 replicate mass spectrometry analyses of each biological replicate pulldown.

Figure S5. Enrichment plots for selected significant (FDR < 1%) interactors identified from IP-MS experiments in pig brain lysates. Each plot reports measured mass spectrometry-measured abundances (colored dots) of the prey protein labeled in the top left of the plot following immunoprecipitation from pig brain (either frontal cortex (FC) or cerebellum (cere), as noted) with antibodies targeting the bait protein labeled on the x-axis, accompanied by data from matched IP experiments using antibodies targeting either GFP or FLAG as negative controls. Abundances are reported in normalized counts per million (cpm) as measured using Degust (see **Methods**) across 3-4 replicate mass spectrometry analyses of each biological replicate pulldown. Note that unlike the case for mouse IPs, the DMXL1 antibody in the pig brain specifically pulls down DMXL2 (bottom row); no DMXL1 is detected in the IPs. DMXL1/2 gene identities were confirmed based on chromosomal synteny.

bioRxiv preprint doi: https://doi.org/10.1101/2025.05.26.656196; this version posted May 28, 2025. The copyright holder for this preprint (which was not certified by peer review) is the author/funder, who has granted bioRxiv a license to display the preprint in perpetuity. It is made available under aCC-BY 4.0 International license.

Figure S6. MA plots for selected IP-MS experiments in mouse whole brain lysates. Each plot reports the log mean abundances (x-axis) versus log ratios (y-axis) of all proteins (colored dots) detected by mass spectrometry at confidence (FDR < 1%) following immunoprecipitation from whole mouse brain with antibodies targeting the bait protein labeled at the top of the plot or by matched IP experiments using antibodies targeting either GFP or FLAG as negative controls. Proteins with positive log ratio values are enriched in the experimental samples; proteins with negative log ratio are enriched in the negative control IPs. Proteins are colored on a blue (non-significant) to red (significantly differentially enriched, FDR < 1%) color scale. Abundances are reported in normalized counts per million (cpm) measured using Degust (see **Methods**) and are matched to the plots in **Figure S4**.

Figure S7. MA plots for selected IP-MS experiments in pig brain lysates. Each plot reports the log mean abundances (x-axis) versus log ratios (y-axis) of all proteins (colored dots) detected by mass spectrometry at confidence (FDR < 1%) following immunoprecipitation from pig brain with antibodies targeting the bait protein (labeled at the top of the plot along with the tissue analyzed) or by matched IP experiments using antibodies targeting either GFP or FLAG as negative controls. Proteins with positive log ratio values are enriched in the experimental samples; proteins with negative log ratio are enriched in the negative control IPs. Proteins are colored on a blue (non-significant) to red (significantly differentially enriched, FDR < 1%) color scale. Abundances are reported in normalized counts per million (cpm) measured using Degust (see **Methods**) and are matched to the plots in **Figure S5**.

bioRxiv preprint doi: https://doi.org/10.1101/2025.05.26.656196; this version posted May 28, 2025. The copyright holder for this preprint (which was not certified by peer review) is the author/funder, who has granted bioRxiv a license to display the preprint in perpetuity. It is made available under aCC-BY 4.0 International license.

SUPPLEMENTAL FIGURES

Figure S1. Alphafold-multimer score distributions for sampled protein pairs as a function of CF-MS interaction score.

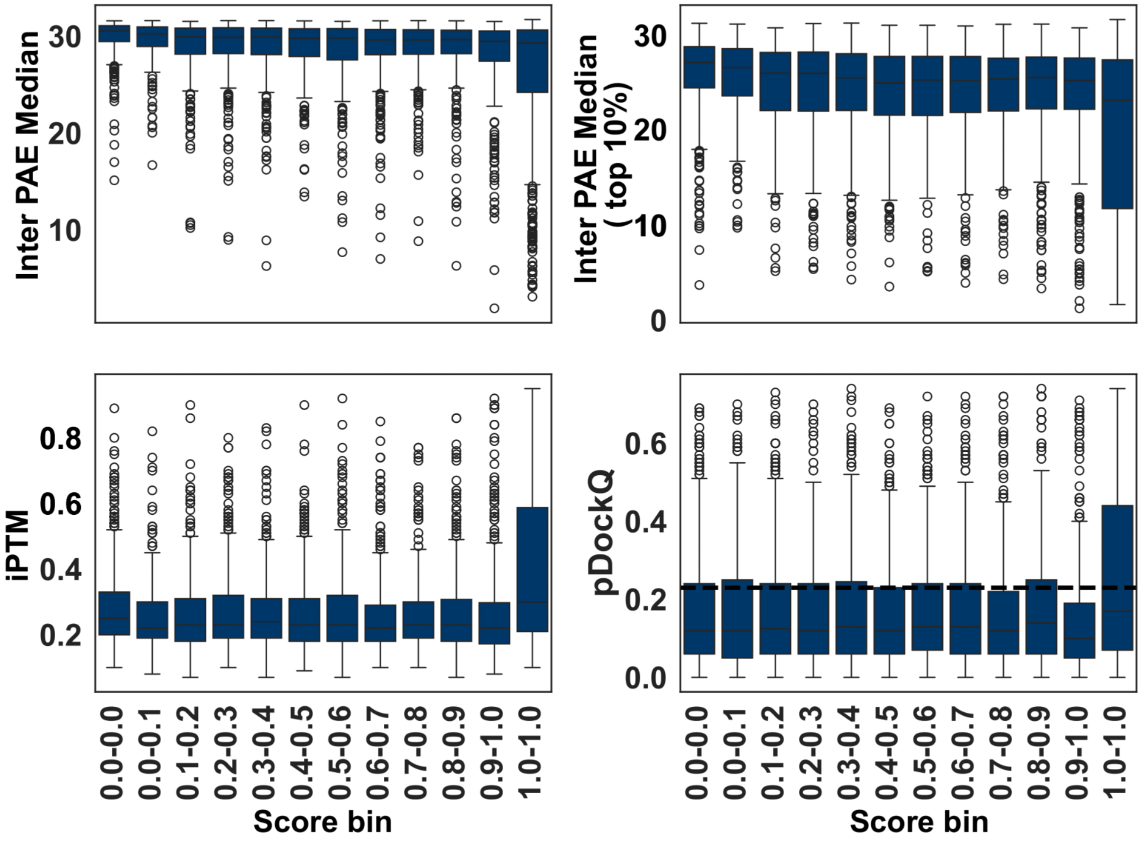

bioRxiv preprint doi: https://doi.org/10.1101/2025.05.26.656196; this version posted May 28, 2025. The copyright holder for this preprint (which was not certified by peer review) is the author/funder, who has granted bioRxiv a license to display the preprint in perpetuity. It is made available under aCC-BY 4.0 International license.

Figure S2. Co-elution profiles show intact subunits of the CCT chaperone and COP9 signalosome complexes from CF-MS experiments in zebrafish brains

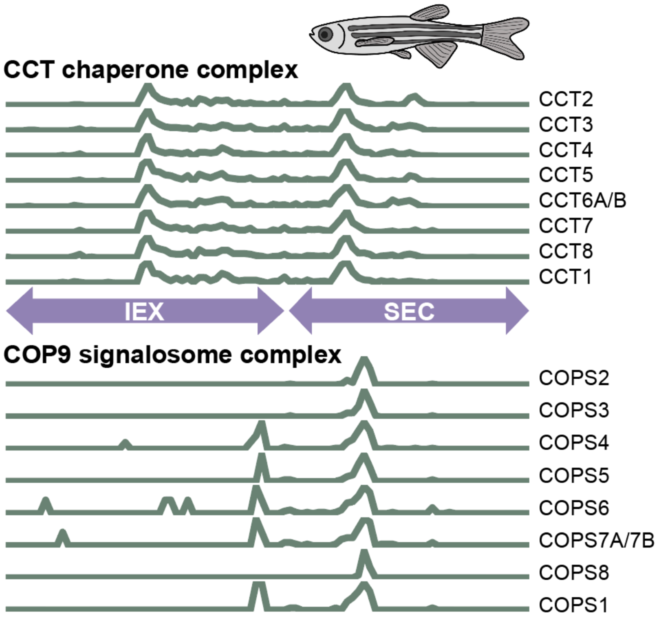

bioRxiv preprint doi: https://doi.org/10.1101/2025.05.26.656196; this version posted May 28, 2025. The copyright holder for this preprint (which was not certified by peer review) is the author/funder, who has granted bioRxiv a license to display the preprint in perpetuity. It is made available under aCC-BY 4.0 International license.

Figure S3. Area under the receiver operating characteristic curve (AUROC) values for correctly associating proteins together in VerteBrain that are known to belong to the same Gene Ontology (GO) Protein containing complex or Cellular anatomical entity.

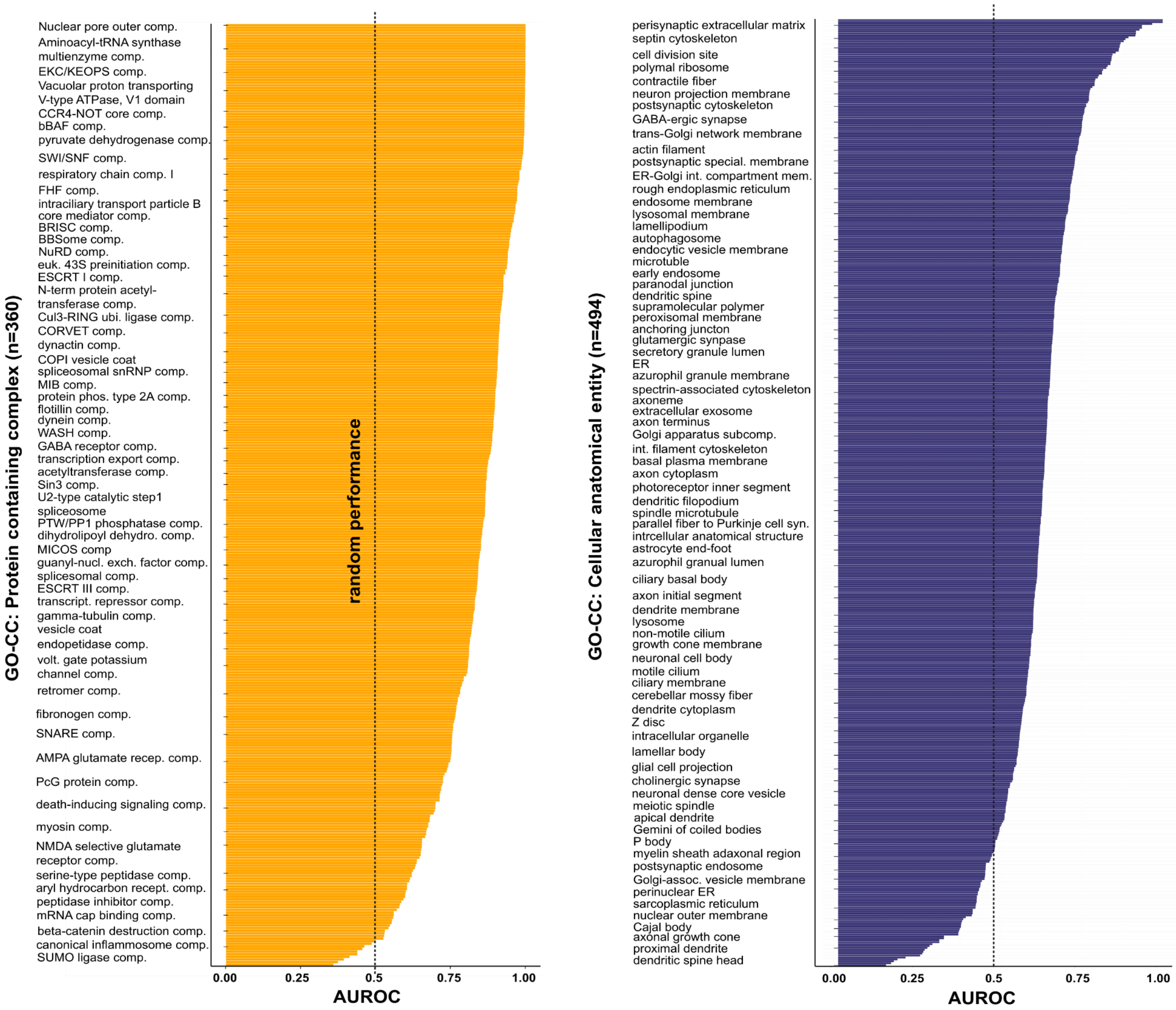

bioRxiv preprint doi: https://doi.org/10.1101/2025.05.26.656196; this version posted May 28, 2025. The copyright holder for this preprint (which was not certified by peer review) is the author/funder, who has granted bioRxiv a license to display the preprint in perpetuity. It is made available under aCC-BY 4.0 International license.

Figure S4. Enrichment plots for selected significant (FDR < 1%) interactors identified from IP-MS experiments in mouse whole brain lysates

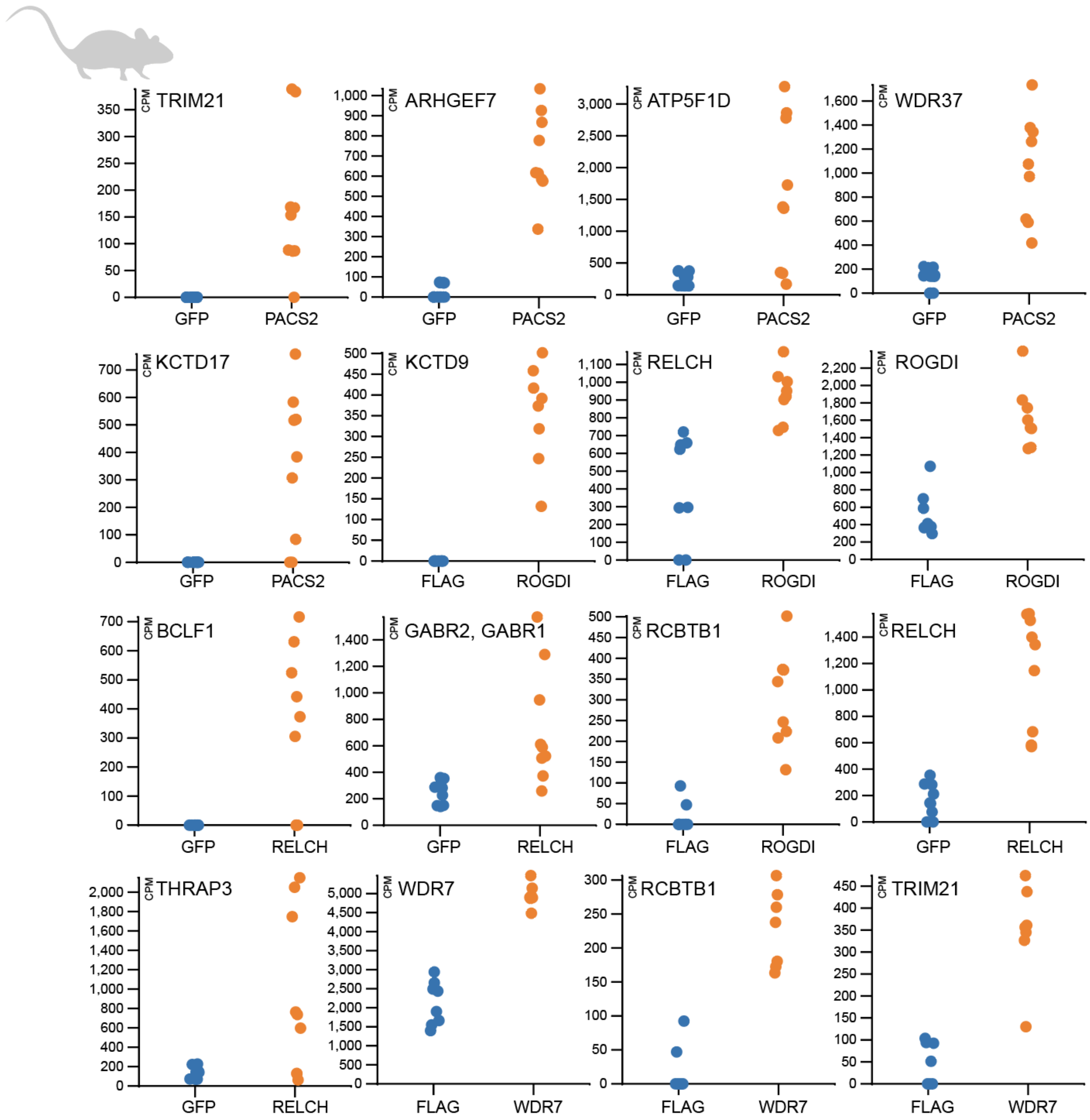

bioRxiv preprint doi: https://doi.org/10.1101/2025.05.26.656196; this version posted May 28, 2025. The copyright holder for this preprint (which was not certified by peer review) is the author/funder, who has granted bioRxiv a license to display the preprint in perpetuity. It is made available under aCC-BY 4.0 International license.

Figure S5. Enrichment plots for selected significant (FDR < 1%) interactors identified from IP-MS experiments in pig brain lysates

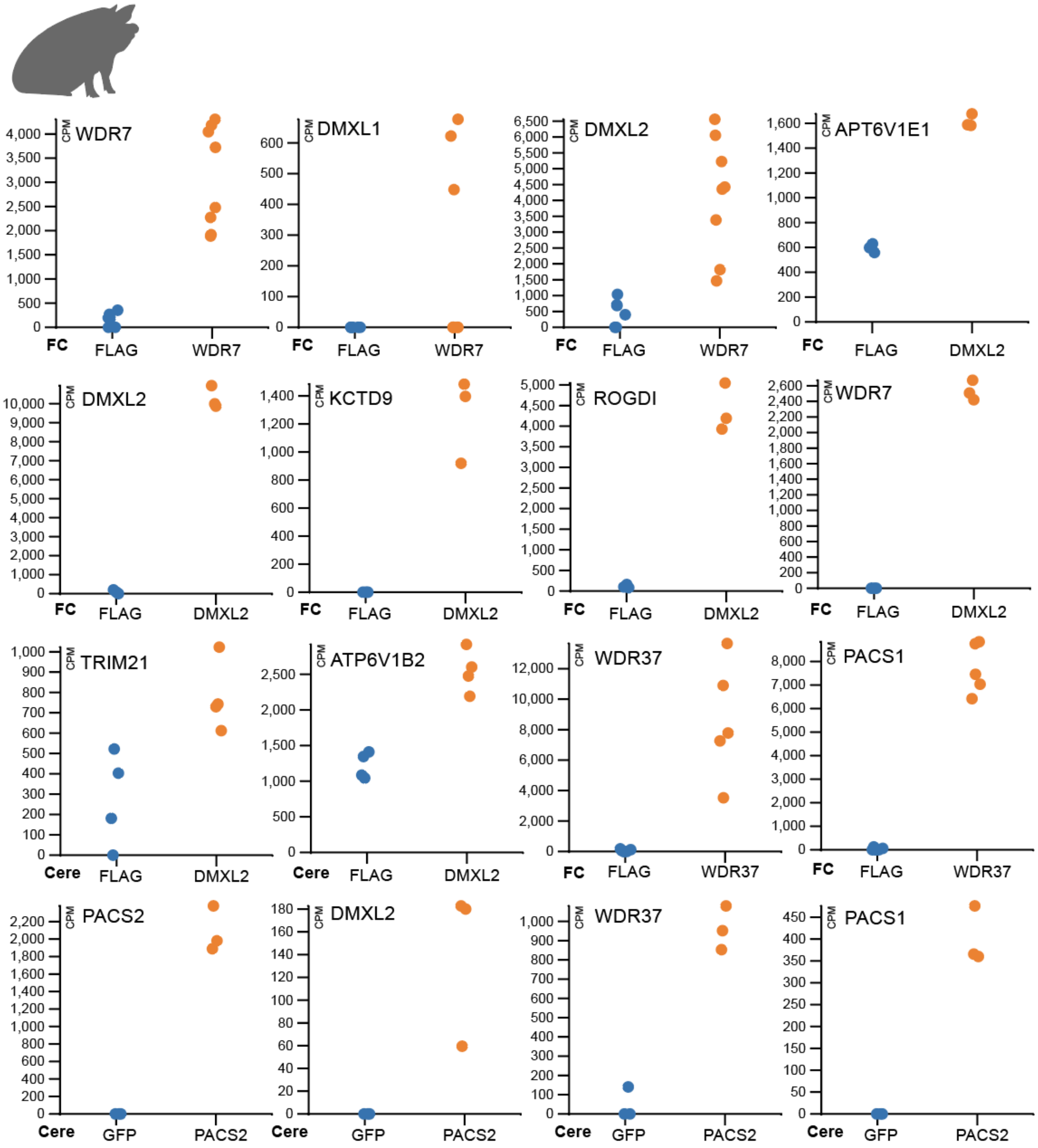

bioRxiv preprint doi: https://doi.org/10.1101/2025.05.26.656196; this version posted May 28, 2025. The copyright holder for this preprint (which was not certified by peer review) is the author/funder, who has granted bioRxiv a license to display the preprint in perpetuity. It is made available under aCC-BY 4.0 International license.

Figure S6A. MA plots for selected IP-MS experiments in mouse whole brain lysates

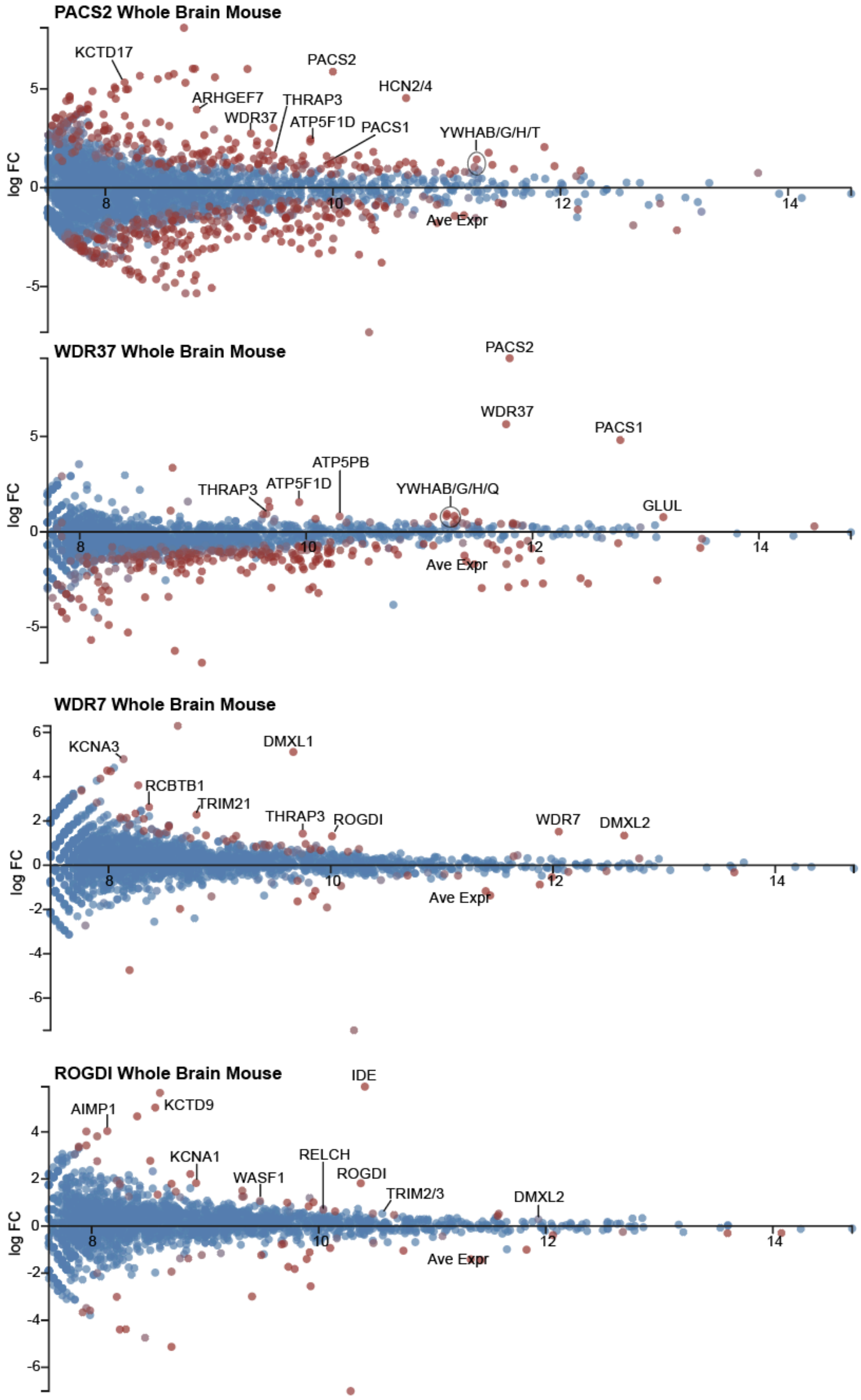

bioRxiv preprint doi: https://doi.org/10.1101/2025.05.26.656196; this version posted May 28, 2025. The copyright holder for this preprint (which was not certified by peer review) is the author/funder, who has granted bioRxiv a license to display the preprint in perpetuity. It is made available under aCC-BY 4.0 International license.

Figure S6B. MA plots for selected IP-MS experiments in mouse whole brain lysates

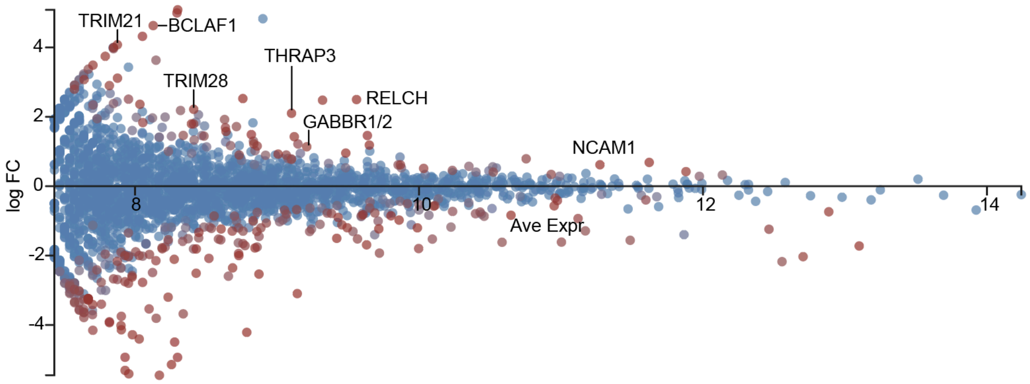

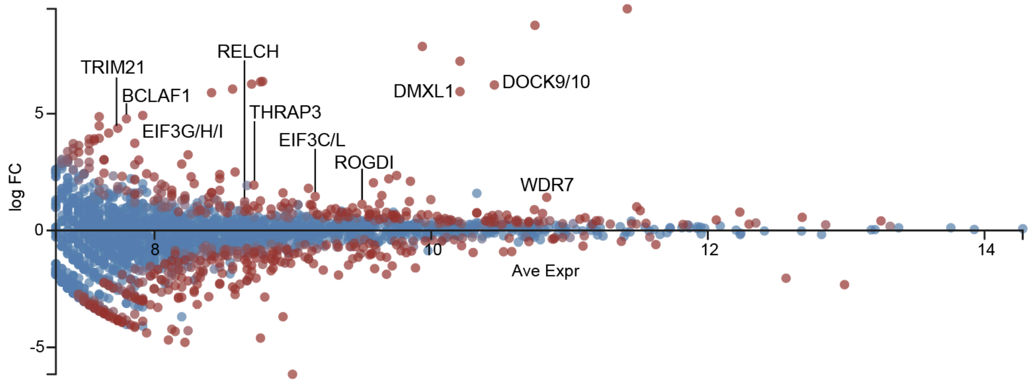

bioRxiv preprint doi: https://doi.org/10.1101/2025.05.26.656196; this version posted May 28, 2025. The copyright holder for this preprint (which was not certified by peer review) is the author/funder, who has granted bioRxiv a license to display the preprint in perpetuity. It is made available under aCC-BY 4.0 International license.

Figure S7A. MA plots for selected IP-MS experiments in pig brain lysates

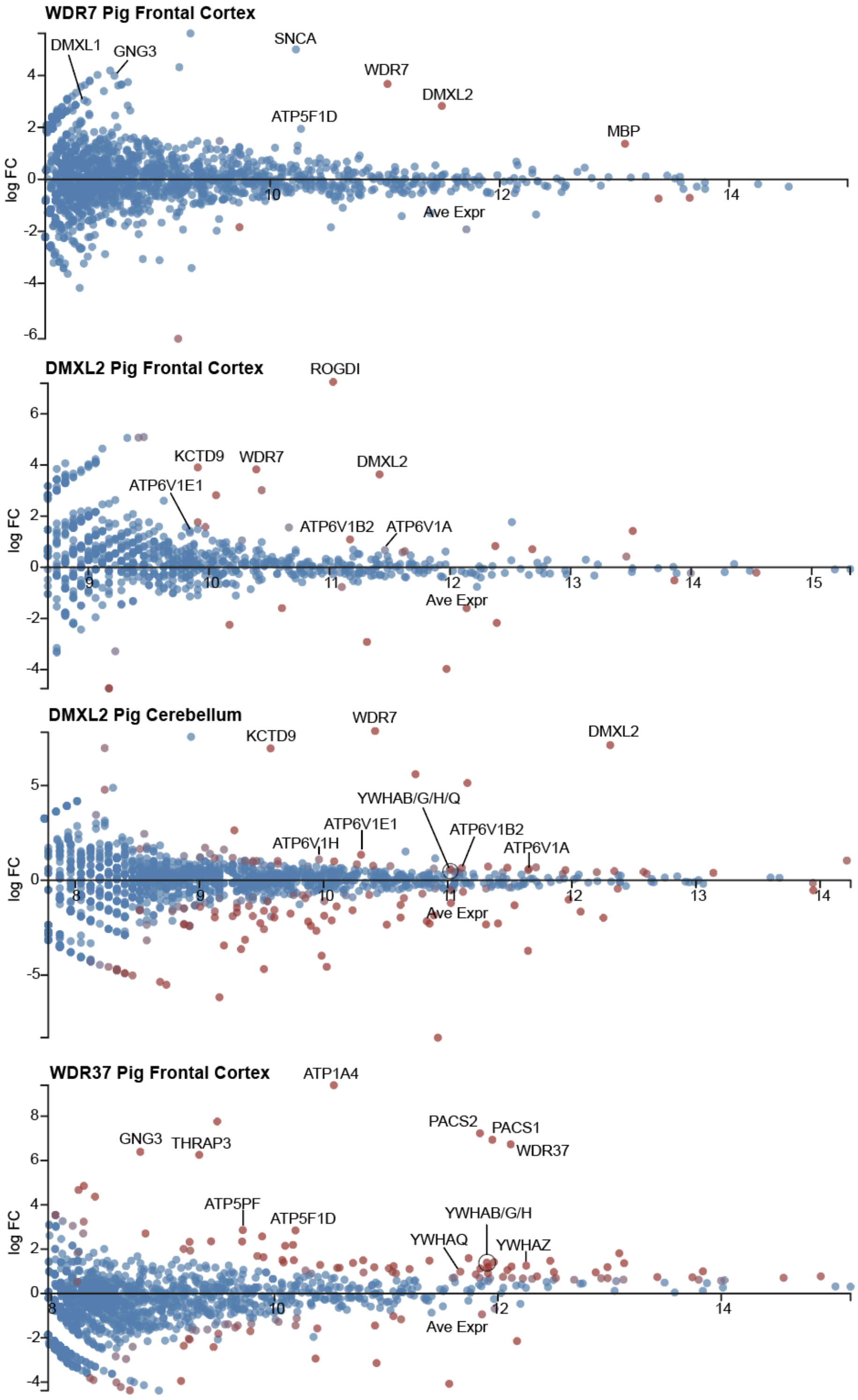

bioRxiv preprint doi: https://doi.org/10.1101/2025.05.26.656196; this version posted May 28, 2025. The copyright holder for this preprint (which was not certified by peer review) is the author/funder, who has granted bioRxiv a license to display the preprint in perpetuity. It is made available under aCC-BY 4.0 International license.

Figure S7B. MA plots for selected IP-MS experiments in pig brain lysates

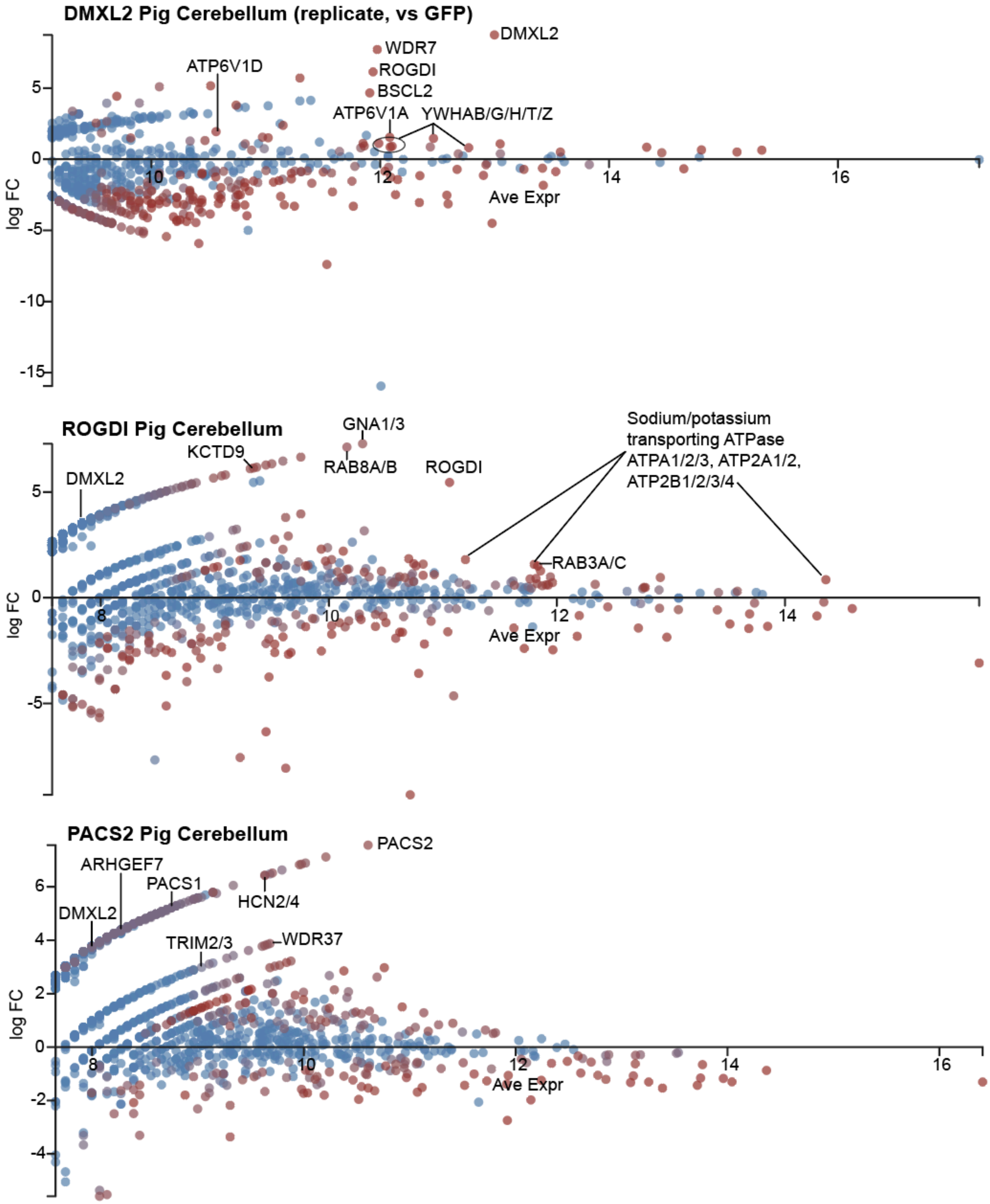

bioRxiv preprint doi: https://doi.org/10.1101/2025.05.26.656196; this version posted May 28, 2025. The copyright holder for this preprint (which was not certified by peer review) is the author/funder, who has granted bioRxiv a license to display the preprint in perpetuity. It is made available under aCC-BY 4.0 International license.

SUPPLEMENTAL TABLES

Table S1: Overview summary of biological samples, reagents, software, and algorithms used in this construct the vertebrate brain interactome.

Table S2: Quantification in peptide-spectral matches of 9,259 verNOG orthogroups across 2,197 biochemical fractions from 35 CF-MS experiments using post-mortem brain tissue samples in five species, related to **Figure 1**.

Table S3: Pairwise interactions in VerteBrain above 8% FDR threshold based on the ExtraTreeClassifier model.

Table S4: 6,108 verNOG orthogroups organized hierarchically into varying granularities of protein assemblies, denoted by the column headers, e.g. as for the column headed “cut_3926”, which provides 3,926 protein assemblies, identified by numerical cluster identifiers.

Table S5: Summary of significant interactors measured by IP-MS of Rabconnectin-3 and WDR37/PACS subunits relative to control IP-MS experiments.
